# Harnessing tetrahedral framework nucleic acids for enhanced delivery of microRNA‐149‐3p: A new frontier in oral squamous cell carcinoma therapy

**DOI:** 10.1111/cpr.13637

**Published:** 2024-04-26

**Authors:** Siqi Xu, Xin Qin, Jiale Liang, Xiao Fu, Dexuan Xiao, Yunfeng Lin, Tao Wang

**Affiliations:** ^1^ Dental Medical Center, Hainan Affiliated Hospital of Hainan Medical University (Hainan General Hospital) Haikou Hainan China; ^2^ State Key Laboratory of Oral Diseases & National Center for Stomatology & National Clinical Research Center for Oral Diseases, West China Hospital of Stomatology Sichuan University Chengdu Sichuan China

## Abstract

Oral squamous cell carcinoma (OSCC), a type of malignant tumour that primarily occurs in the oral mucosa, has drawn considerable attention owing to its aggressive growth and potentially high metastatic rate. Surgical resection is the primary treatment method for OSCC and is typically combined with radiation therapy and chemotherapy. microRNA‐149‐3p (miR‐149) is a negative regulator of the Pi3k/Akt pathway and can effectively inhibit the proliferation of tumour cells. However, the application of miR‐149 is limited owing to its relatively low efficiency of cellular uptake and poor stability when used alone. To overcome these challenges, this study adopted a novel nucleic acid nanostructured material, tetrahedral framework nucleic acids (tFNAs). The use of tFNAs as carriers to assemble the T‐miR‐149 complex reduced the expression of Pi3k and Akt involved in tumorigenesis and alterations in proteins related to cell apoptosis. The results indicated that the bionic drug delivery system has an effective tumour suppressive effect on OSCC in mice, revealing its potential clinical value in the treatment of OSCC.

## Graphical abstract

The use of tFNAs as carriers to assemble the T‐miR‐149 complex reduced the expression of Pi3k and Akt involved in tumorigenesis and alterations in proteins related to cell apoptosis. The bionic drug delivery system has an effective tumour suppressive effect on OSCC in mice, revealing its potential clinical value in the treatment of OSCC.

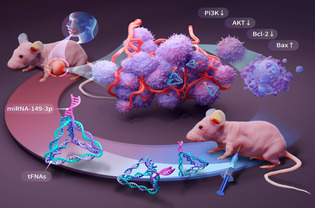

## 1 INTRODUCTION

Oral squamous cell carcinoma (OSCC) is a common malignant epithelial tumour of the oral maxillofacial region accounting for up to 90% of the tumour cases. The consumption of tobacco and alcohol, as well as Betel nuts, is considered a potential risk factor contributing to the high incidence of OSCC. However, the 5‐year survival rate of patients with OSCC is relatively low, often less than 60%.^1^ Surgical resection is the preferred treatment for early‐stage OSCC, later‐stage patients rely on a combination of radiotherapy and chemotherapy to control tumour progression. Current chemotherapy regimens include various types of drugs such as antimetabolites (e.g., methotrexate and 5‐fluorouracil), microtubule inhibitors (e.g., paclitaxel), platinum compounds (e.g., cisplatin) and antitumor antibiotics (e.g., bleomycin and doxorubicin).^2^ However, increased drug resistance or immune tolerance of tumour cells has resulted in unsatisfactory treatment effects. Further, because these treatments indiscriminately target both normal and tumour cells, they may damage the surrounding healthy organs, leading to severe side effects.^3^ Therefore, new treatment methods that are safe, effective and less toxic to healthy tissues must be developed.^4^

MicroRNAs (MiRs, miRNAs), strands of non‐coding RNA molecules that are about 18–23 nucleotides long, play a key role in human cancer development by regulating over 60% of the protein‐coding genes.^5^ As part of an important regulatory RNA family, they interact with a large subset of target gene spectra, influencing nearly all complex genetic networks and cellular signalling cascades, with even a single miRNA being capable of regulating a wide range of targets.^6^ They are closely related to the aetiology, progression and prognosis of cancer by regulating tumour cell apoptosis, differentiation, invasion and metastasis. These are the potential therapeutic targets for the clinical treatment of cancer. MicroRNA‐149‐3p (miR‐149) is a recently discovered member of the MiR family.^7^ In the human genome, miR‐149 is located on chromosome 11 in the q13.1 region. MiR‐149 is often considered a tumour suppressor, exhibiting inhibitory effects in cancers, such as oesophageal cancer and OSCC.^8^ As a negative regulator of the Pi3k/Akt signalling pathway, miR‐149 significantly inhibits tumour cell proliferation, rendering it a potential gene therapy strategy. Due to inherent biosafety issues, miRNA therapy continues to face challenges in the preclinical stage. Associated obstacles include the inability of miRNA to passively diffuse through lipid membranes and the lack of a reliable miRNA delivery system. Further research is needed to unveil an optimized, efficient and low‐toxicity miRNA delivery system that can effectively deliver exogenous miRNA into cells to exert its intended function.^9^

Tetrahedral framework nucleic acids (tFNAs) are a new type of nanonucleic acid with tremendous potential for drug and gene delivery.^10^ By editing DNA sequences and lengths, a three‐dimensional tetrahedral structure can be synthesized, characterized by excellent compatibility and highly sequence‐conserved. The essence of tFNAS is nucleic acid, which, compared to traditional carriers and cationic particles, may offer better biocompatibility and lower immunogenicity. Their unique three‐dimensional structure facilitates outstanding cellular penetration capabilities, allowing them to easily traverse cell membranes and conveniently carry drugs and therapeutic molecules into target cells.^11^ Moreover, tFNAs can be edited to target tumour cell nuclear sequences, thereby enhancing miRNA targeting and reducing off‐target effects in vivo. Furthermore, tFNAs are more stable than traditional single‐ or double‐stranded nucleotides in serum and can escape from lysosomes through nuclear localization signals, thus ensuring efficient gene delivery.

This study constructed a complex based on the tFNAs system loaded with miR‐149, named T‐miR‐149. T‐miR‐149 protected miR‐149 from degradation and stably entered the system, effectively inhibiting the invasion and migration of Cal27 cells.^12^ Additionally, the potential cellular pathways affected by T‐miR‐149, particularly the Pi3k and Akt pathways, were investigated to lay the groundwork for subsequent in vivo experiments. Using tFNAs as carriers, along with miR‐149, significantly improves the overall survival rate of mice.^13^ Thus, this may have a profound impact on the future use of T‐miR‐149 as an antitumor drug and could represent an advanced method to improve the survival rate of patients with cancer.^14^

## 2 RESULTS AND DISCUSSION

### 2.1 Preparation and Characterization of tFNAs and T‐miR‐149

**FIGURE 1:**
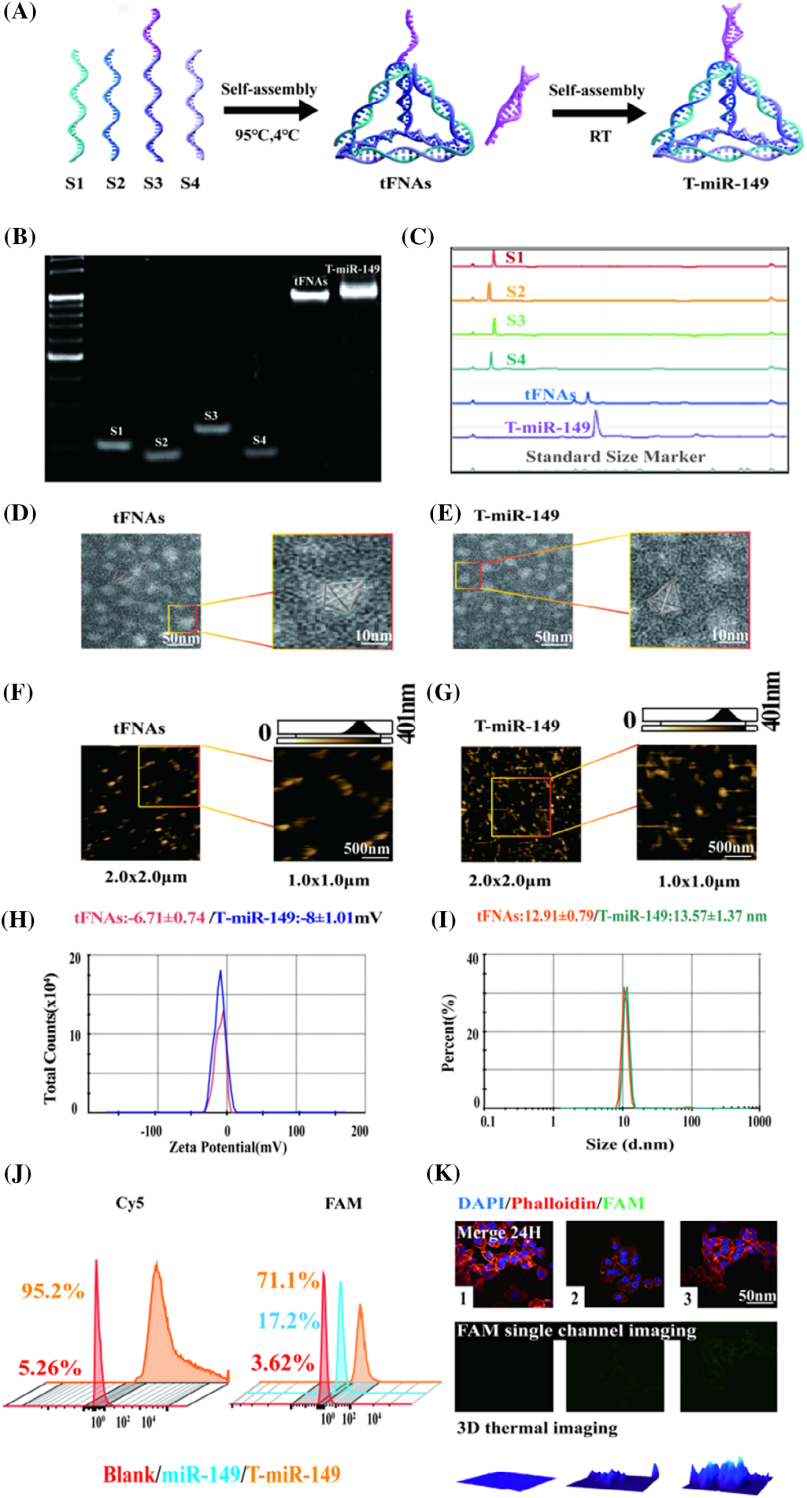
(A) Schematic of the sticky‐ended tetrahedral framework nucleic acids (tFNAs) and T‐miR‐149 assembly. (B) Polyacrylamide gel electrophoresis image showing the successful synthesis of tFNAs and T‐miR‐149. (C) Confirmation of successful synthesis of tFNAs and T‐miR‐149 based on high‐performance capillary electrophoresis. (D,E) Transmission electron microscopy images of tFNAs and T‐miR‐149. Scale bars represent 50 and 10 nm. (F,G) Atomic force microscopy images of the tFNAs and T‐miR‐149. Scale sizes were 1 and 500 nm. (H) Zeta potentials of tFNAs and T‐miR‐149 measured by dynamic light scattering (DLS). (I) Molecular sizes of tFNAs and T‐miR‐149, as determined by DLS. (J) Flow cytometric analysis of Cal27 cell uptake of FAM‐T‐miR‐149 after 24 h. (K) Confocal fluorescence microscopy analysis verifying the cellular entry performance of Fam‐T‐miR‐149 and Fam‐miR‐149 (green: Fam; blue: Nucleus; red: Cytoskeleton; scale bar: 50 μm).

tFNAs can be readily assembled from four ssDNAs based on the principle of base complementary pairing with the specific base sequences shown in Table S1. In this study, the method of connecting miR‐149 to tFNAs was similar to that used in previous studies. A sticky end was attached to the 5′ end of the S3 strand, which paired with the complementary sticky end of miR‐149, thus completing the synthesis of T‐miR‐149.^15^ A schematic of the synthetic process is presented in Figure 1A. To ensure successful synthesis, polyacrylamide gel electrophoresis (PAGE) was performed to detect differences in molecular weight. T‐miR‐149, was loaded with miR‐149 and had a larger molecular weight, and moved at a slower speed during gel electrophoresis compared with tFNAs (Figure 1B). These attributes were confirmed by high‐performance capillary electrophoresis (Figure 1C). In addition to the characterization of the physicochemical properties of T‐miR‐149, atomic force microscopy (AFM) and transmission electron microscopy (TEM) were used to observe its morphology.^16^ TEM revealed the tetrahedral structure of tFNAs (Figure 1D,E), while AFM revealed that miR‐149 was clearly attached to the apex of tFNAs (Figure 1F,G). Dynamic light scattering (DLS) was used to explore the differences in particle size and zeta potential between tFNAs and T‐149‐3p. Because T‐miR‐149 carried miR‐149, it had a larger particle size and more negative zeta potential than tFNAs. After loading miR‐149, the particle size of the T‐miR‐149 complex increased from 12.91 to 13.57 nm, (Figure 1H), and the zeta potential shifted from −6.71 to −8 mV (Figure 1i). Thus, we successfully synthesized T‐miR‐149. The tFNAs delivery system maintains the stability of miR‐149 against degradation and efficiently transports miR‐149 into cells because of its unique three‐dimensional structure. Previous studies have shown that tFNAs can enter cells via clathrin‐mediated endocytosis via cell‐surface receptors.^17^ The ordinary miR‐149 strand was replaced with a Fam‐labelled miR‐149 strand to monitor the entry of T‐miR‐149 into cells (Figure 1J). CLSM confirmed the penetration of T‐miR‐149, with a gradually increasing Fam fluorescence intensity inside the cells, whereas only faint fluorescence was observed in the miR‐149 group (Figure 1K). Overall, these results demonstrate the successful synthesis of T‐miR‐149 and confirm its good biocompatibility in Cal27 cells, as well as its superior capability as an miRNA therapeutic drug carrier (Figure S1).^18^

### 2.2 The cytotoxic T‐miR‐149 suppresses cell migration

**FIGURE 2:**
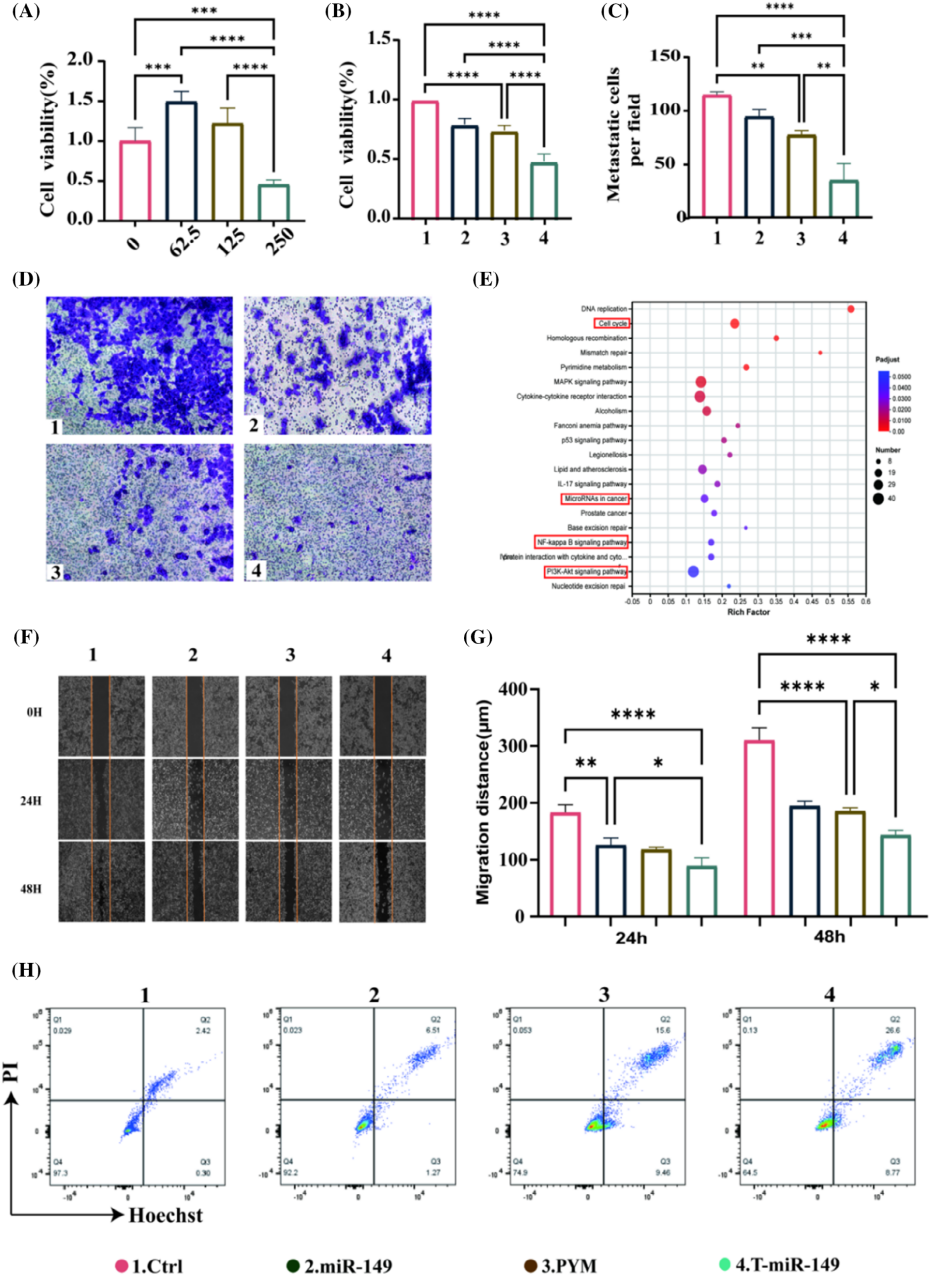
(A) Effects of different concentrations of T‐miR‐149 on the cell viability of Cal27. (B) Assessment of cell viability in different treatment groups. (C) Statistical graph of the transwell experiment. (D) Cal27 cell invasion ability in a transwell experiment observed with polyformaldehyde‐fixed crystal violet staining. (E) Top 20 significant enrichment pathways diagram. The horizontal axis represents the enrichment factor. (F) Images of wound healing assay see the horizontal migrating ability of Cal27 after different treatments within 48 h. (G) Statistical graph of the scratch assay. (H) Flow cytometric analysis of the percentage of apoptosis and necrosis in Cal27 cells across different treatment groups. All data are presented as means ± standard deviations (SD) (*n* = 3) and analysed using a one‐way analysis of variance followed by Tukey's multiple comparisons tests. Statistical significance is indicated by *p < 0.05, **p < 0.01, ***p < 0.001 and ****p＜0.0001.

Interactions between T‐miR‐149 and Cal27 cells are essential for suppressing migration. In this study, CCK8 experiments were performed to detect cell viability. The cytotoxic effect of T‐miR‐149 was examined, and 250 nmol/L was found to be the optimal concentration for the in vitro cellular model of T‐miR‐149 (Figure 2A).^19^ Accordingly, we selected these as the optimal concentrations for subsequent experiments. Following cell grouping and treatment, T‐miR‐149 significantly inhibited the viability of Cal27 cells compared with groups treated with miR‐149 and PYM (Figure 2B). Microscopic observations and transwell assays showed fewer cells migrating to the upper chamber in the T‐miR‐149 group.^20^ In contrast, a considerable increase in cell migration was observed in the control group, indicating that the inducer treatment significantly inhibited tumour cell invasion (Figure 2C,D). This is an important pathway diagram between the Ctrl and T‐miR‐149 groups. The larger the horizontal axis, the greater the enrichment degree. The size of the points indicates the number of transcripts, and the colour of the points corresponds to different ranges, transitioning from blue to red to indicate a sequential decrease in the p‐value (Figure 2E). Wound healing assays demonstrated a significant difference between the control and T‐miR‐149 groups in terms of wound closure (*p* < 0.01), indicating a significant effect of T‐miR‐149 on the migratory ability of Cal27 cells (Figure 2F,G). Flow cytometry was used to determine the effect of T‐miR‐149 on apoptosis.^21^ The high apoptotic rate in the T‐miR‐149 group (26.6%) indicated that T‐miR‐149 significantly induced apoptosis in Cal27 cells. The inhibitory effects of Ctrl (2.42%), miR‐149 (6.51%) and PYM (15.6%) increased successively, and the effects of PYM were significantly stronger than those of Ctrl and miR‐149. The PYM group exhibited the highest necrotic rate (9.46%), and the necrotic rate of T‐miR‐149 (8.7%) was only slightly lower than that of the PYM group.^22^ Thus, T‐miR‐149 significantly enhanced apoptosis in Cal27 cells compared with other treatment groups.^23^ They may augment the pro‐apoptotic effects of miR‐149 through a synergistic action, thus indicating that T‐miR‐149 is a promising apoptotic agent (Figure 2H).

### 2.3 T‐miR‐149 promotes apoptosis‐related proteins

**FIGURE 3:**
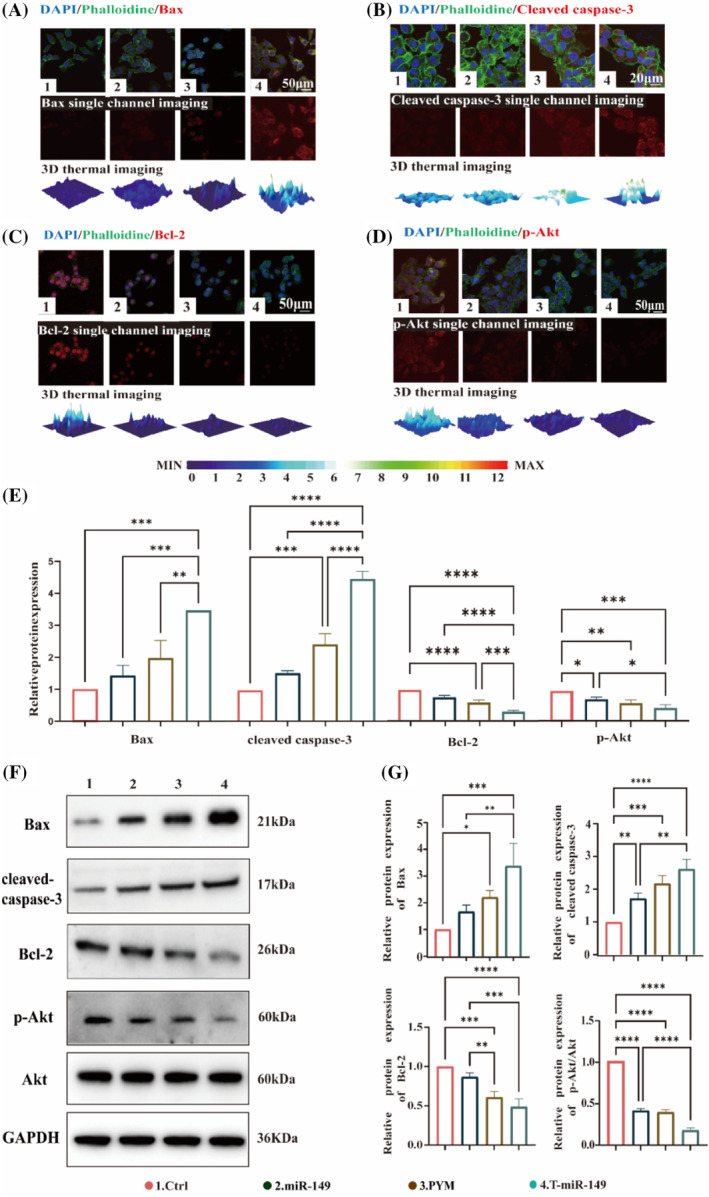
(A–D) Immunofluorescence images showing the expression of Bax, cleaved caspase‐3, Bcl‐2 and p‐Akt in Cal27 cells 24 h after different treatments (Bax, cleaved caspase‐3, Bcl‐2 and p‐Akt: red; cytoskeleton: green; nuclei: blue; 3D heat imaging: reconstructs the intensity of protein fluorescence; scale for cleaved caspase‐3: 20 μm; scale for Bax, Bcl‐2 and p‐Akt: 50 μm). (E) Quantitative immunofluorescence. (F) Western blotting results for the expressions of Bax, cleaved caspase‐3, Bcl‐2 and p‐Akt. (G) Quantitative analysis of the Western blotting results. All data are presented as means ± SD (*n* = 3) and were analysed using a one‐way analysis of variance followed by Tukey's test for multiple comparisons. Statistical significance is indicated by *p < 0.05, **p < 0.01, ***p < 0.001 and ****p＜0.0001.

Apoptosis is a programmed cell death process controlled by genes that maintain a balance in the cellular internal environment. The Akt/Bcl‐2 signalling pathway is a typical apoptotic pathway wherein the Bcl‐2 family of proteins plays a crucial role in regulating cellular survival and death.^24^ Bcl‐2 is an anti‐apoptotic protein that inhibits caspase‐3‐mediated cellular apoptosis, with caspase‐3 serving as an essential executor protein during the apoptotic process. Conversely, protein kinase B (Akt) is a specific protein kinase that must be activated through phosphorylation and aids in maintaining cell survival.^25^ Akt increases the activity of anti‐apoptotic components of the Bcl‐2 family (such as Bcl‐2), thereby inhibiting apoptosis. However, intervention with T‐miR‐149 shifted this balance, thus weakening the inhibitory effect of Akt on pro‐apoptotic components (such as Bax) and increasing apoptosis. To study in‐depth the potential regulatory mechanism of T‐miR‐149 in this process, immunofluorescence experiments were conducted; these showed that T‐miR‐149 inhibited the expression of Bcl‐2 and p‐Akt, whereas the levels of Bax and cleaved‐caspase3 were increased (Figure 3A–E). Western blotting was used to detect the expression of the Akt/Bcl‐2 pathway‐related proteins Bax, cleaved‐caspase3, Bcl‐2 and p‐Akt (Figure 3F,G). The T‐miR‐149 group had the highest expressions of Bax and cleaved‐caspase3 proteins compared with those of the MiR149 and PYM groups, thus suggesting that T‐miR‐149 was involved in apoptosis.^26^ Thus, the apoptotic stimulatory effect of T‐miR‐149 on Cal27 cells may inhibit the Pi3k/Akt pathway, down‐regulate Akt expression and consequently enhance the apoptotic differentiation specificity.

### 2.4 T‐miR‐149 enhance antitumor efficacy

**FIGURE 4:**
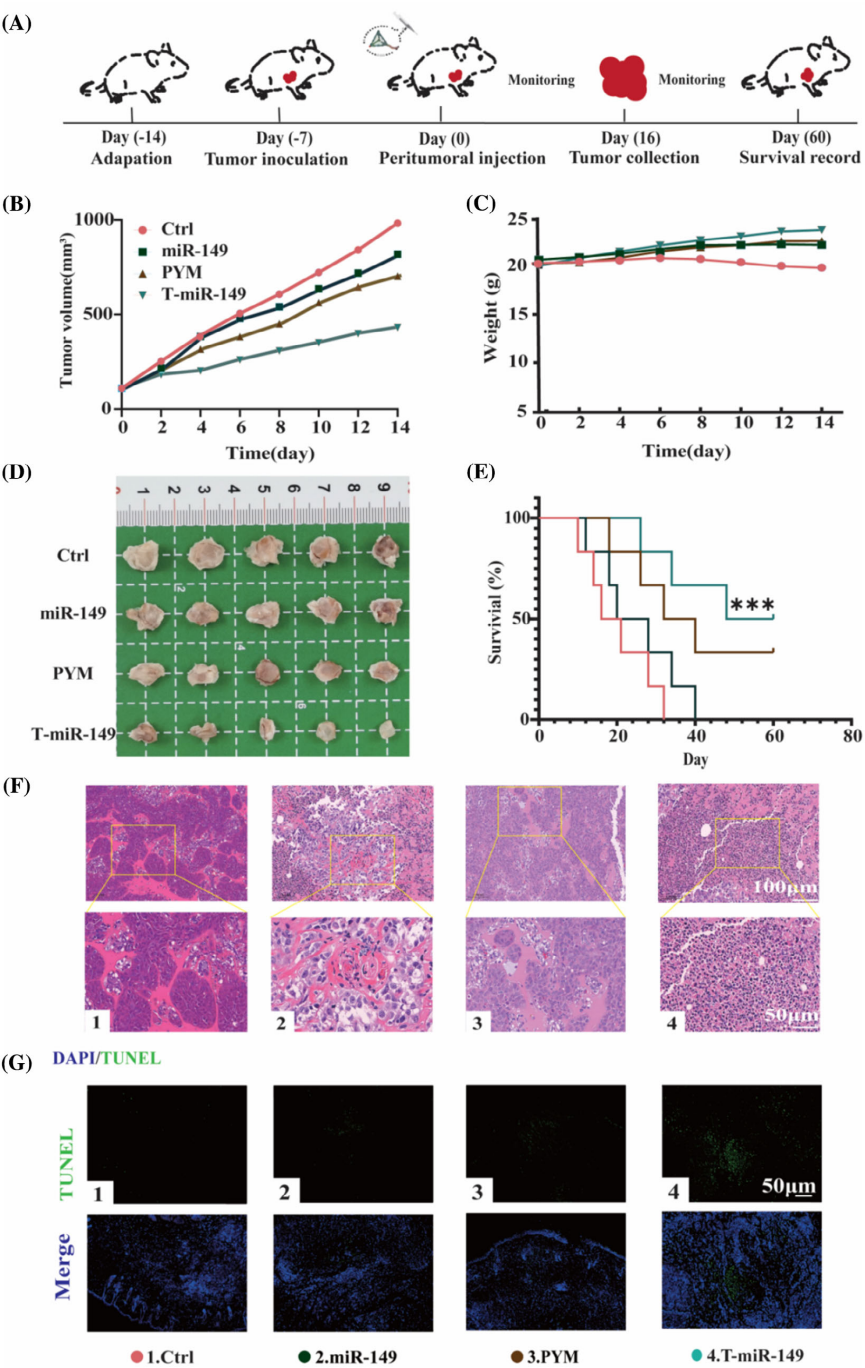
(A) Schematic representation of in vivo antitumor experiments in tumour‐bearing mice. (B) Tumour‐bearing mice received local injections every 2 days, and tumour volumes were recorded during the experiment. (C) Changes in the tumour weights of mice in each group. (D) Tumours were excised from tumour‐bearing mice and photographed at the end of the experiment (*n* = 5). (E) Mouse survival rate. (F) HE staining of collected tumours (scale bar: 50 μm). (G) TUNEL staining of collected tumours (blue: cell nuclei, green: apoptotic cells; scale bar: 50 μm). Data are presented as means ± SD (*n* = 5); statistical significance: ***p < 0.001.

To investigate the in vivo effects of T‐miR‐149, we established an animal model using BALB/c nude mice. The pre‐treated Cal27 cells were locally injected into the mice's axillary region.^27^ Synthetic drugs were locally administered to the tumour via injections every 2 days (Figure 4A), while recording the tumour volume and mouse weight. Figure 4C illustrates the trends in body weight changes among different groups of mice during the administration period.^28^ One week after tumour inoculation, the following observations were made: mice in the phosphate‐buffered saline (PBS) group exhibited a gradual decrease in body weight, whereas mice in the miR‐149 and PYM groups experienced slower rates of weight gain. In contrast, the mice in the T‐miR‐149 group exhibited a normal increase in body weight.^29^ This pattern is consistent with the characteristic weight loss observed in patients during the tumour development period; in the PBS‐treated group, tumours grew rapidly, while tumour growth was inhibited by miR‐149, PYM and T‐miR‐149 treatment (Figure 4B). The order of subcutaneous tumour size is as follows: PBS > miR‐149 > PYM > T‐miR‐149, indicating that T‐miR‐149 has the stronger anti‐tumour ability (Figure 4D). The median survival time of T‐miR‐149‐treated mice exceeded 60 days, with 35% of the PYM‐treated mice surviving for 60 days. In contrast, 50% of the mice treated with MiR149 survived for 20–28 days (Figure 4E). Tumour sections from each treatment group exhibited nuclear pleomorphism, with the MiR149 and PYM treatment groups showing abundant pleomorphic nuclei, whereas the T‐miR‐149 treatment group had fewer nuclear heterogeneities (Figure 4F).^30^ Furthermore, both the PYM and T‐miR‐149 groups showed evidence of partial necrotic tissue, which was verified by TUNEL staining. To stain the nuclei of tumour cells, 4′,6‐diamidino‐2‐phenylindole (DAPI) was used,^31^ making them appear as distinct blue. Concurrently, the TUNEL‐positive fluorescent signal in the cells is shown in green. The percentage of TUNEL‐positive cells relative to the total cell count and the relative fluorescence intensity of TUNEL were used as measurement standards to quantify the degree of cell apoptosis.^32^ In the PBS‐treated group, apoptotic cells were almost nonexistent, whereas in the T‐miR‐149‐treated group, the highest number of apoptotic cells was observed. Similarly, compared with the miR‐149 and PYM treatment groups, the TUNEL fluorescence intensity in the PBS group was the weakest, whereas the T‐miR‐149 group showed the most significant fluorescence intensity, indicating a more prominent therapeutic effect (Figure 4G).

### 2.5 miR‐149 inhibited Pi3k/Akt pathway in vivo

**FIGURE 5:**
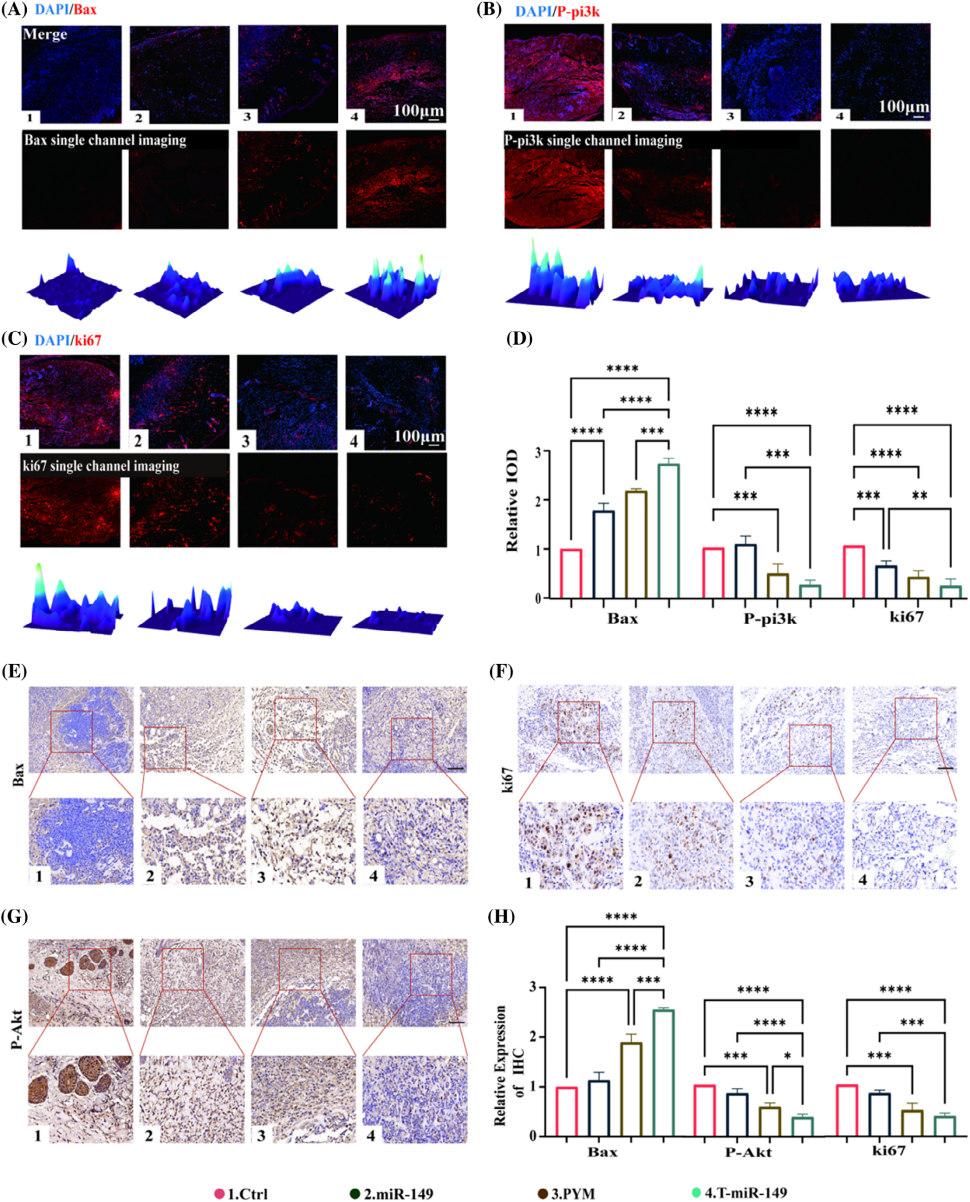
(A–C) Immunofluorescence images showing the expressions of P‐pi3k, Ki67 and Bax (P‐pi3k, Ki67 and Bax: red; nuclei: blue; 3D heat maps: fluorescence intensity reconstruction of P‐pi3k, Ki67 and Bax; scale bar: 100 μm). (D) Quantitative analysis of relative fluorescence intensities of P‐pi3k, Ki67 and Bax. (E–G) Immunohistochemical images depicting Bax, Ki67 and p‐Akt expression (scale bar: 50 μm). (H) Quantitative analysis based on the immunohistochemical images of the proportion of cells positive for Bax, Ki67 and p‐Akt. All data were analysed using one‐way analysis of variance and post‐hoc analysis and are presented as means ± SD (*n* ≥ 3). Error bars represent SD.

To confirm the role of T‐miR‐149 in the antitumor mechanism in OSCC, we evaluated it using immunohistochemistry and immunofluorescence staining methods. In terms of immunofluorescence staining (Figure 5A–D), we quantitatively analysed the expression levels of Bax, Pi3k and Ki67 by measuring the intensity of green fluorescence.^33^ Ki67 protein is a key tumour marker used to indicate cellular proliferation processes. The expression level of Ki67 is a crucial indicator for assessing the proliferation rate of tumour cells,^34^ with high Ki67 expression typically associated with accelerated cell division and significantly enhanced proliferative capacity. Additionally, changes in Ki67 expression levels were used to monitor the effectiveness of treatment^35^; for instance, a decrease in Ki67 levels often indicates positive therapeutic outcomes. The Bax protein exhibited the highest expression level in the T‐miR‐149 group, showing an upward trend in all groups except for the PBS group, with T‐miR‐149 demonstrating the strongest protein fluorescence expression.^36^ Additionally, the trends of p‐Pi3k and Ki67 proteins were similar; both exhibited lower positive expressions in the PYM and T‐miR‐149 groups and the lowest in the T‐miR‐149 group. Regarding immunohistochemistry (Figure 5E–H), the expression levels of p‐Akt, Bax and Ki67 in tumours were measured by calculating the proportion of positively stained cells, with positive staining visualized as areas with brownish‐yellow colours in the cytoplasm or cell nuclei.^37^ For the Ki67 protein. Most cell nuclei in the PBS‐treated group showed positive staining, indicating active proliferation, whereas the proportion of positively stained nuclei decreased in the other treatment groups (Figure 5H). The findings suggested that T‐miR‐149 had a significant antitumor effect superior to miR‐149 and PYM, thereby confirming the in vitro experimental results.^38^ Tumour necrosis factor alpha can directly target specific groups of tumour cells, inducing their apoptosis or necrosis. Additionally, it can catalyse the activity of immune cells, such as macrophages and T cells,^39^ thereby enhancing their destructive capabilities against tumour cells. Interferon‐gamma is a potent immunomodulator and can activate natural killer cells,^40^ thereby strengthening their ability to attack and eliminate tumour cells (Figure S2). Histopathological examination of important organs in mice.^41^ Haematoxylin and Eosin (HE) staining of important organs did not reveal pathological injury, indicating that T‐miR‐149 has good biocompatibility and biosafety characteristics in vivo (Figure S3).

## 3 CONCLUSIONS

The experiments suggested a connection between the occurrence and development of OSCC and the Pi3k/Akt pathway. Compared with existing chemotherapeutic drugs, treatment with T‐miR‐149 is more precise and associated with fewer side effects.^42^ To slow the progression of OSCC, we introduced tFNAs as a favourable nucleic acid carrier of miR‐149 and successfully constructed a novel nanocomplex (T‐miR‐149).^43^ This complex exhibited outstanding drug delivery and cellular entry capabilities, significantly improving drug release compared with free miR‐149.^44^ Cell experiments revealed that T‐miR‐149 significantly enhanced the apoptosis‐promoting ability of free miR‐149 in tumour cells through the Pi3k/Akt and Akt/Bcl‐2 pathways,^45^ and in vivo experiments further confirmed the antitumor efficacy of T‐miR‐149. The initiation of DNA gene medicine has led to the emergence of more effective novel DNA gene materials, aiming to enhance the efficacy of cancer treatment and potentially play pivotal roles in other biological applications in the future. Throughout the research process, T‐miR‐149 has demonstrated encouraging biological safety and significant anti‐tumour effects.^46^ The first‐time modification of miR‐149 onto DNA nanomaterials not only enhanced tumour‐targeting capabilities but also facilitated the lysosomal degradation of biomarker proteins. Therefore, tFNAs are a promising drug delivery carrier for miR‐149, providing new insights and new opportunities for the treatment of OSCC. Considering the high editability of tFNAs, future research could explore the synthesis of new tFNAs/miR‐149 complexes by modifying the targeting aptamers.^47^

## 4 EXPERIMENTAL SECTION

### 4.1 Materials

See supporting information for details.

### 4.2 Synthesis and Identification of tFNAs and T‐miR‐149

A solution containing 10 mM Tris–HCl and 50 mM MgCl at pH 8.0 was used as the synthesis buffer (TM buffer). Single‐stranded DNAs (ssDNAs) were added to the TM. The system was heated to 95°C and then annealed at 4°C to prepare tFNAs. Subsequently, miR‐149 mimic was mixed with S‐tFNA to synthesize T‐miR‐149. The materials were stored long‐term at 4°C. tFNAs/T‐miR‐149 was validated using PAGE and DLS techniques. The morphological characteristics and approximate size of T‐miR‐149 were observed using TEM and AFM.

### 4.3 Cell culture and xenograft animal models

The human OSSC cell line CAL27 was cultured as follows. Initially, the cells were cultured in a DMEM/HIGH medium (Gibco) supplemented with 10% fetal bovine serum (Zeta Life, Australia) and 1% penicillin/streptomycin (Gibco). The cells were then incubated in a 5% CO_2_ incubator at 37°C. Healthy 4–5‐week‐old male BALB/c nude mice were xenografted with Cal27 cells to establish an OSCC disease model. Log‐phase cells were first digested with trypsin and prepared as single‐cell suspensions in PBS. The cell concentration was adjusted to 2 × 10^6^ cells/mL, and 0.2 mL of the cell suspension was injected subcutaneously into the right forelimbs of the nude mice. The mice were subsequently housed in specific pathogen free animal facilities.

### 4.4 Cellular uptake of T‐miR‐149

In this study, the ability of tFNAs and T‐miR‐149 to enter Cal27 cells was tested. One of the ssDNAs in the tFNAs was modified with fluorescein labelling, and the modified T‐miR‐149 was added to the adherent cells. After 24 h, the fluorescence intensity was measured using a flow cytometer and compared with the blank control group. Further, the intracellular uptake of T‐miR‐149 was observed using confocal laser scanning microscopy. The treated cells were fixed in 4% paraformaldehyde. Finally, the cell nuclei and membrane were stained with DAPI and red Phalloidin, respectively, for identification.

### 4.5 Assessment of cell viability

Cells were seeded in a 96‐well plate at a density of 5 × 10^3^ cells per well and incubated overnight at 37°C in a 5% CO_2_ environment. Subsequently, the cells were exposed to the blank, miR‐149, PYM and T‐miR‐149 groups for 24 h. Thereafter, 90 μL of the culture medium and 10 μL of CCK8 solution were added to each well for further incubation. After incubation, the 96‐well plate was placed in a microplate reader, and the absorbance of each group was measured at a wavelength of 450 nm.

### 4.6 Scratch assay

First, the lines were marked on the back of a 6‐well culture plate. Approximately 2 × 10^5^ cells per well were seeded, and a scratch was made the following day. The dislodged cells were washed and divided into groups for drug treatment. Photographs were captured under a 40× microscope at 0, 24 and 48 h after culture. The mean distance between cells was calculated using the ImageJ software.

### 4.7 Analysis of cell apoptosis

After cells were treated with T‐miR‐149, cells were collected by trypsin digestion at specific time points. The samples were then centrifuged to remove the supernatant. The cell pellet was resuspended in 0.8–1 mL of cell staining buffer. Thereafter, 5 μL of Hoechst stain and 5 μL of PI stain were added and thoroughly mixed. The mixture was incubated at 4°C in the dark for 20 min. Finally, flow cytometry was performed to observe the changes in cell apoptosis.

### 4.8 Immunofluorescence assay

See supporting information for details.

### 4.9 Western blot

See supporting information for details.

### 4.10 Statistical analysis

All the experiments were repeated at least thrice. The data are presented as the mean value and its standard deviation. Student's *t* test or one‐way analysis of variance (ANOVA) was applied for comparing the groups. When the *p*‐value was less than 0.05, we considered the difference between the groups to be statistically significant.

## AUTHOR CONTRIBUTIONS

Siqi Xu, Xin Qin as well as Jiale Liang conducted the majority of the experiments, receiving assistance from Xiao Fu, Dexuan Xiao performed the experiments involving animals. Tao Wang and Yunfeng Lin supervise the project.

## FUNDING INFORMATION

This work was supported by Hainan Province Science and Technology Special Fund, Grant/Award Number: ZDYF2021SHFZ114; This study was supported by the National Natural Science Foundation of China (Grant No. 81960199). Supported by Clinical Translational Innovation Cultivating Fund 550 Project of Hainan General Hospital. Supported by Joint Program on Health Science & Technology Innovation of Hainan Province (Grant/Award No. SQ2023WSJK0557).

## CONFLICT OF INTEREST STATEMENT

The authors declare no competing financial interest.

## Supporting information


**Data S1:** Supporting Information.

## Data Availability

The data that support the findings of this study are available from the corresponding author upon reasonable request.
