# Supplementary material for: Harnessing tetrahedral framework nucleic acids for enhanced delivery of microRNA‐149‐3p: A new frontier in oral squamous cell carcinoma therapy
Source: Cell Prolif. 2024 Apr 26;57(8):e13637. doi: 10.1111/cpr.13637 (PMC11294420; doi:10.1111/cpr.13637)
Supplement: Supplementary file 1 — Data S1: Supporting Information. [file CPR-57-e13637-s001.docx]

**Supporting Information**

**Harnessing Tetrahedral Framework Nucleic Acids for Enhanced Delivery of microRNA-149-3p: A New Frontier in Oral Squamous Cell Carcinoma Therapy**

Siqi Xu^1†^, Xin Qin^2†^, Jiale Liang^2^, Xiao Fu^1^, Dexuan Xiao^2^, Yunfeng Lin^2^*, Tao Wang^1^*

^1^ Dental Medical Center, Hainan, Affiliated Hospital of Hainan Medical University (Hainan General Hospital), 19 Xiuhua Road, Haikou, Hainan 570311, China

^2^ State Key Laboratory of Oral Diseases & National Center for Stomatology & National Clinical Research Center for Oral Diseases, West China Hospital of Stomatology, Sichuan University, Chengdu 610041, Sichuan, China

^†^These authors contributed equally to this work.

***Corresponding Author Emails:**

Tao Wang: [18608917377@163.com](mailto:18608917377@163.com)

Yunfeng Lin: [yunfenglin@scu.edu.cn](mailto:yunfenglin@scu.edu.cn)

# Experimental section

## Materials

The Cal27 cell line was obtained from the American Type Culture Collection (ATCC). In this study, 5- week-old male BALB/c nude mice were provided by Chengdu Ensworth Biotechnology Co., Ltd. (Chengdu, China). DMEM/high glucose culture medium, 0.25% trypsin solution, and penicillin streptomycin solution were all purchased from Gibco. The 10% fetal bovine serum (FBS) was obtained from Zet a Life (Australia). Four single-stranded DNA and microRNA-149-3p were purchased from Shanghai Sangon Biotech Co., Ltd. The following items were purchased from Bi Yun Tian Company: Cell apoptosis and necrosis assay kit, Cell Counting Kit-8 (CCK-8). Transwell chambers were purchased from Cornin g (New York, USA), and ELISA-related reagents were obtained from Shanghai Zhuocai Biotechnology Co., Ltd. Antibodies used for protein determination were purchased from Abcam (Cambridge, U.K.) and CST (Qingdao, China). The antibodies used in this study are listed in Table S2.

## Western Blot

To further measure the changes in proteins related to cell apoptosis (Bcl-2, p-Akt, Bax and Cleaved Caspase-3), Western blotting was conducted. Cells were cultured in 6-well plates, and protein was extracted from the treated Cal27 cells using the whole-cell lysis assay method. Proteins were separated using sodium dodecyl sulfate-PAGE, cut from the gel, and then transferred onto a polyvinylidene fluoride membrane. They were then transferred to a membrane under constant current. Subsequently, the membrane was blocked using a QuickBlock™ blocking buffer and then incubated overnight with the appropriate primary antibodies at 4 °C. The next day, after returning to room temperature, the membranes were incubated for 1 h with secondary antibodies at a dilution of 1:3000. After washing with the TBST buffer, immunoblotting was conducted, and images were captured to observe changes in protein expression. GAPDH was used as an internal control, and ImageJ software was used for the quantitative analysis of protein expression.

## Immunofluorescence Assay

Cells were seeded in confocal dishes after 24 h of culture and then grouped for drug treatment. The next day, the cell samples were fixed with 4% paraformaldehyde, permeabilized with 0.5% Triton X-100, and blocked with 5% goat serum. Primary antibodies were incubated overnight at 4 °C. On the following day, after returning to room temperature, the samples were incubated with secondary antibodies. The cell cytoskeleton and nuclei were stained with phalloidin conjugated to a green fluorescent dye and DAPI, respectively. After washing with PBS, the expression levels of the relevant proteins were observed using confocal laser scanning microscopy.

## ELISA Assay

Following equilibration of the reagent kit at room temperature for 30 min, different concentrations of the standard were added to the standard wells. The test sample was added to the sample wells, and no substances were added to the blank wells. Following enzymatic treatment, the reaction system was incubated in a constant-temperature incubator at a set temperature for 60 min. After incubation, the washing solution was discarded and the wells were blotted dry with absorbent paper. Washing buffer was thoroughly added to each well and allowed to stand briefly, and the washing steps were repeated. Subsequently, the substrate solution was added, and the reaction system was incubated at 37 °C in the dark. After adding the stop solution, the optical density (OD) was measured at a wavelength of 450 nm.

# Supporting Tables

**Table S1. The sequences of four ss DNAs composing T and T-miR-149**

| **ss DNA** | **Base sequence** | **Direction** |
| --- | --- | --- |
| S1 | ATTTATCACCCGCCATAGTAGACGTATCACCAGGCAGTTGAGAC GAACATTCCTAAGTCTGAA | 5’→3’ |
| S2 | ACATGCGAGGGTCCAATACCGACGATTACAGCTTGCTACACGA TTCAGACTTAGGAATGTTCG | 5’→3’ |
| S3 | TTGACCTGTGAATTACTACTATGGCGGGTGATAAAACGTGTAGCAAG CTGTAATCGACGGGAAGAGCATGCCCATCC | 5’→3’ |
| S4 | ACGGTATTGGACCCTCGCATGACTCAACTGCCTGGTGATACGAGGAT GGGCATGCTCTTCCCG | 5’→3’ |
| microRNA-149-3pmimic sense | UUCACAGGUCAAAGGGAGGGACGGGGGCUGUGC | 5’→3’ |
| microRNA-149-3pmimic antisense | CCCUAUCACAAUUAGCAUUAA | 5’→3’ |

**Table S2. Antibodies applied for Western Blot (WB) and immunofluorescence (IF) in this paper**

| **Antibodies** | **Company** | **Application** | **Dilution fold** |
| --- | --- | --- | --- |
| GAPDH | Cell Signaling Technology | WB | 1:1000 |
| Phospho-Akt | Cell Signaling Technology | WB | 1:1000 |
| Akt | Cell Signaling Technology | WB | 1:2000 |
| Bcl-2 | Abcam | WB | 1:1000 |
| Bax | Abcam | WB | 1:1000 |
| Anti-cleaved caspase-3 | Abcam | WB | 1:500 |
| Phospho-Akt | Abcam | IF | 1:400 |
| Bax | Abcam | IF | 1:100 |
| Bcl-2 | Abcam | IF | 1:200 |
| Anti-cleaved caspase-3 | Abcam | IF | 1:250 |

# Supporting Figures

**
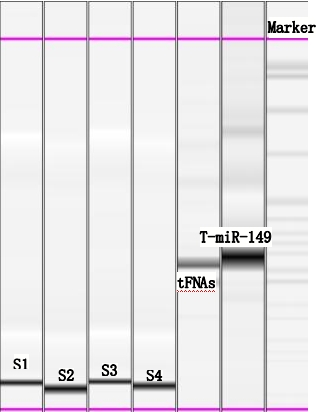
**

**Fig. S1** Identification of ssDNA and the successful synthesis of tFNAs and T-miR-149 through high-performance capillary electrophoresis experiment.


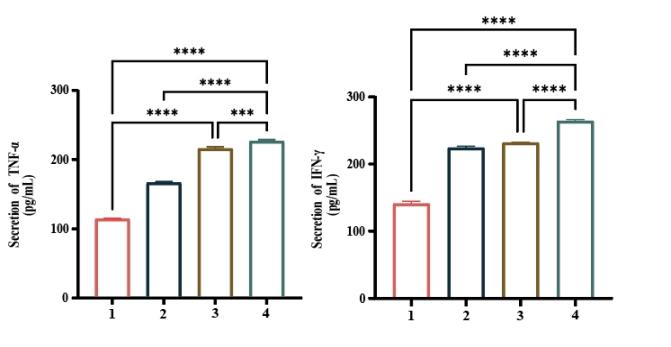


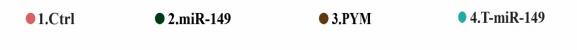


**Fig. S2** Statistical analysis of the expression of mouse TNF-α and IFN-γ by ELISA (n = 5).


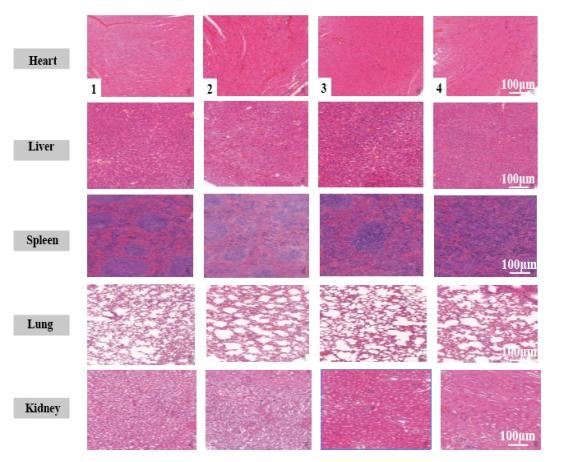


**Fig. S3** H&E staining of hearts, livers, spleens, lungs and kidneys. Scale bars are 100μm.
